# Unlocking zeolite-like structures as a new family of interstitial oxide ion conductors: insights into carrier trapping, collective local distortion, and correlated disorder†

**DOI:** 10.1039/d5sc02898a

**Published:** 2025-07-16

**Authors:** Xianyi Wei, Xiaohui Li, Aydar Rakhmatullin, Xiaoge Wang, Cheng Li, Hankun Xu, Sihao Deng, Lunhua He, Kun Lin, Qiang Li, Junliang Sun, Xianran Xing, Xiaojun Kuang

**Affiliations:** a Guangxi Key Laboratory of Electrochemical and Magnetochemical Functional Materials, College of Chemistry and Bioengineering, Guilin University of Technology Guilin 541004 People's Republic of China xiaohuili@glut.edu.cn kuangxj@glut.edu.cn; b Conditions Extremes et Materiaux: Haute Temperature et Irradiation (CEMHTI-CNRS), CEMHTI UPR3079, University of Orléans F-45071 Orléans France; c College of Chemistry and Molecular Engineering, Peking University, Beijing National Laboratory for Molecular Science (BNLMS) Beijing 100871 People's Republic of China; d Oak Ridge National Laboratory, Neutron Sciences Directorate Oak Ridge TN 37831-2008 USA; e Beijing Advanced Innovation Center for Materials Genome Engineering, Institute of Solid State Chemistry, University of Science and Technology Beijing Beijing 100083 People's Republic of China; f Songshan Lake Materials Laboratory Dongguan 523808 China; g Spallation Neutron Source Science Center Dongguan 523803 People's Republic of China; h Beijing National Laboratory for Condensed Matter Physics, Institute of Physics, Chinese Academic of Sciences Beijing 100190 People's Republic of China

## Abstract

Zeolites have emerged as indispensable materials for applications ranging from catalysis and separation to adsorption and ion exchange, owing to their uniquely ordered porous architectures composed of well-defined channels and cavities. Inspired by the promising oxygen defect tolerance observed in various open structural frameworks, herein, we have developed a new zeolite-like feldspar structure, A_2_B_2_C_2_O_8_ with 4- and 8-membered rings, in the case of Sr_1−*x*_La_*x*_Ga_2_Ge_2_O_8+0.5*x*_, as a new family of interstitial oxide ion conductors due to its open structural framework for accommodating and transporting interstitial oxide ions. Average structural analysis revealed that the interstitial oxygen occupies the centers of 4-membered rings, existing in a coordination equilibrium quasi-free state that confers high mobility; however, this contrasts with the experimentally observed low mobility. Local structural analysis further revealed that the local collective distortions in GaO_4_ and GeO_4_ tetrahedra, together with the correlated disorder of interstitial oxygen coordinated with Ge, hidden within the average structure, are critical to interstitial oxygen mobility from the 4-membered ring to the 8-membered ring. Our findings demonstrate zeolite-like structures as a new family of interstitial oxide ion conductors, offering new insights into the intricate interplay between oxide ion mobility, collective distortions, and correlated disorder at the local scale.

## Abstract 2

This work demonstrates zeolite-like structures as a new family of oxide ion conductors, bridging the gap between local structure and ionic migration, providing a foundation for designing ion-conducting materials *via* local structural modifications.

## Graphical abstract

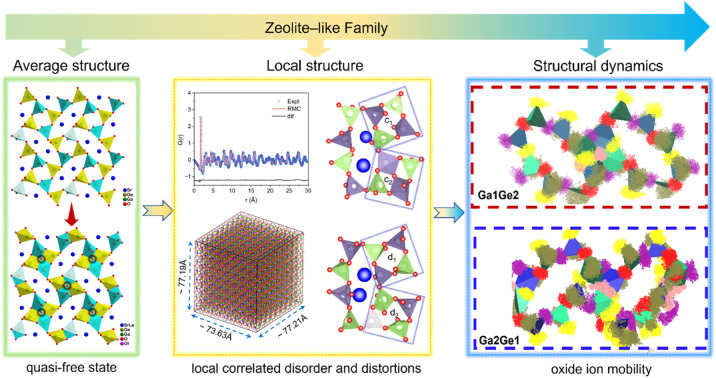

## 1. Introduction

Solid oxide fuel cells (SOFCs) are emerging as a transformative platform for electrochemical energy conversion, directly converting the chemical energy of fuels into electricity with high efficiency, fuel flexibility, and environmental friendliness.^1–3^ Central to their functionality are oxide ion conductors, which act as electrolytes for transporting oxide ions in SOFCs and also have environmental applications in a wide range of devices such as oxygen separation membranes, pumps, and sensors.^4–7^ In order to reduce the cost and enhance long-term stability for these practical applications, it is highly desirable to lower their operating temperatures in the future. The transport of oxide ions in oxide ion conductors, which is primarily governed by oxygen vacancies or interstitial defects that provide pathways for ionic migration, plays a critical role in determining the operating temperatures. However, developing high-performance oxide ion conductors operating at low temperature through tailoring the compositions, defects and structures remains a great challenge, particularly in balancing high oxide ion conductivity with chemical and mechanical stability under operating conditions.^8,9^

To address these challenges, various structural prototypes have been developed successively as candidates for oxide ion conductors. These structures are either linked polyhedral networks or isolated polyhedral anion structures, which include the three-dimensional fluorite,^10^ pyrochlore,^11^ perovskite,^12^ langasite structures,^13^ as well as the low-dimensional structures containing tetrahedral units, such as LAMOX (La_2_Mo_2_O_9_),^14^ scheelite,^15^ apatite,^16^ melilite-type structures,^17^ and borate*-*based oxide ion conductors, (Gd/Y)_1−*x*_Zn_*x*_BO_3−0.5*x*_.^18^ Summarizing the structural features and oxide ion conductivities of the above oxide ion conductors reveals that materials with open structural frameworks show remarkable potential for accommodating oxygen defects with high mobility. Among these open frameworks, the melilite and langasite families are notably two classical open structural types owing to their remarkable defect-tolerant properties.^13,17,19,20^

**Fig. 1:**
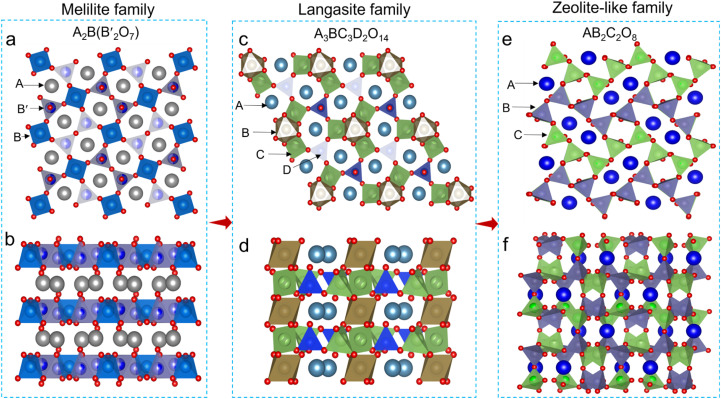
Typical open structural frameworks. (a and b) Melilite, (c and d) langasite and (e and f) zeolite-like feldspar structures viewed along the (a, b and e) [001] and (b, d and f) [100] directions.

The melilite family (A_2_B(B′_2_O_7_)) features a two-dimensional (2D) layered structure composed of corner-sharing tetrahedral anionic layers (BO_4_ with 4 bridging oxygens and B′O_4_ with 3 bridging and 1 terminal oxygen) that form 5-membered rings accommodating the large A cations (Fig. 1a and b).^21^ By tuning the cationic composition and introducing excess positive charge, interstitial oxide ions can be stabilized within the pentagonal rings between A cations in melilite structures. Notably, the typical melilite La_1.54_Sr_0.46_Ga_3_O_7.27_ (ref. 17) demonstrates exceptionally high interstitial oxide ion conductivity (∼0.02 S cm^−1^ at 600 °C), surpassing those of most commonly used electrolytes below 600 °C, with the interstitial oxygen defect migrating *via* a concerted “knock-on” mechanism involving interstitial and lattice oxygen.^22^ In contrast, the langasite family adopts a trigonal structure (A_3_BC_3_D_2_O_14_, Fig. 1c and d),^23–25^ composed of layers of four-coordinated CO_4_ tetrahedra (with 4 bridging oxygens) and three-coordinated DO_4_ tetrahedra (with 3 bridging and 1 terminal oxygen), interconnected by BO_6_ octahedra. This topology imparts exceptional compositional flexibility to the langasite structure. Rosseinsky *et al.*^19^ showed that the langasite structure can accommodate high concentrations of interstitial oxygen, although the interstitial oxygens are trapped within the 6-membered rings with limited mobility. These structural commonalities highlight the potential of open structural frameworks as a promising direction for developing advanced oxide ion conductors.^13^

Driven by the promise of open structural framework architectures, the zeolite-like family could emerge as a compelling candidate for advanced oxide ion conductors. Zeolites are inorganic crystalline materials constructed from corner-sharing TO_4_ tetrahedra (T = Si, Al, P, *etc.*), forming precisely ordered microporous structures.^26^ This unique architecture forms uniform channels and cavities that confer exceptional (hydro)thermal stability, a unique porous framework, and tunable acid sites. These features make zeolites indispensable in a wide range of applications, including catalysis, separation, adsorption, and ion exchange.^27^ Beyond these traditional applications, zeolites have recently shown remarkable promise in energy storage systems. They were initially employed as functional layers on separators or electrodes and as additives in composite solid electrolytes for lithium-ion batteries. However, their potential in ion transport has not yet been adequately recognized.^28–30^ More recently, Yu *et al.*^31^ reported a zeolite X (LiX) membrane serving as the solid electrolyte with superior environmental adaptability and electrochemical stability, addressing critical limitations of conventional solid electrolytes for Li–air batteries. The unique open structural features and outstanding properties in ion transport and chemical stability indicate that zeolite-based materials could be developed into oxide ion conducting electrolytes for advancing the SOFC technology. So far, the research into their role in oxide ion transport remains in its infancy.

Building on this concept, we explore a zeolite-like feldspar structure with the general formula AB_2_C_2_O_8_, where A represents an alkaline earth metal (*e.g.*, Ca^2+^ or Sr^2+^ or Ba^2+^), B is a trivalent metal (*e.g.*, Al^3+^ or Ga^3+^), and C is a tetravalent metal (*e.g.*, Si^4+^ or Ge^4+^) (Fig. 1e and f). This zeolite-like structure, composed of 4- and 8-membered rings,^32^ holds great promise for accommodating and transporting interstitial oxide ions. Herein, the zeolite-like case, strontium feldspar SrGa_2_Ge_2_O_8_, was identified as a potential interstitial host due to its open structural framework and the variable coordination numbers of Ga and Ge that facilitate oxygen defect accommodation and migration. The interstitial oxygen defects were created by La-doping at the Sr site in SrGa_2_Ge_2_O_8_ and the average structure of Sr_1−*x*_La_*x*_Ga_2_Ge_2_O_8+0.5*x*_ containing interstitial oxygen was determined by neutron powder diffraction (NPD). X-ray and neutron pair distribution function (PDF) analysis based on reverse Monte Carlo (RMC) modeling, combined with 1D and 2D ^71^Ga solid state NMR technique, extended X-ray absorption fine structure (EXAFS) analysis, and molecular dynamics (MD) simulations, further revealed that the local collective distortion and correlated disorder in the local structure, hidden within the average structure, are intricately associated with interstitial oxygen mobility. This study provides critical insights into the potential of the zeolite-like family as a new class of oxide ion conductors.

## 2. Experimental section

### 2.1 Synthesis method

Polycrystalline samples of La_*x*_Sr_1−*x*_Ga_2_Ge_2_O_8+0.5*x*_ were synthesized using a conventional high-temperature solid-state reaction method. High-purity raw materials SrCO_3_ (99.9%, Aladdin), La_2_O_3_ (99.99%, Aladdin), Ga_2_O_3_ (99.9%, Aladdin), and GeO_2_ (99.99%, Aladdin) were weighed according to the desired stoichiometries and mixed thoroughly in ethanol using an agate mortar and pestle. The mixed powders were pressed uniaxially into pellets with a diameter of 10 mm and a thickness of approximately 1 mm. The pellets were sintered at 1100 °C for 20 h in air, with heating and cooling rates of 5 °C min^−1^, obtaining a relative density of approximately 90%.

### 2.2 Characterization

Phase formation in the samples was examined by powder X-ray diffraction (XRD) using a Panalytical X'Pert PRO diffractometer with a PIXcel 1D detector using Cu Kα radiation. Variable-temperature XRD patterns were collected on this instrument equipped with an Anton Paar HTK 1200N high temperature attachment. Time-of-flight (TOF) neutron powder diffraction (NPD) data were collected from a general purpose powder diffractometer (GPPD) at the China Spallation Neutron Source (CSNS).^33^ The total scattering neutron pair distribution function (nPDF) data were collected on the BL-1B NOMAD beamline at the Spallation Neutron Source (SNS), Oak Ridge National Laboratory. Average lattice fitting was conducted using PDFgui software.^34^ Reverse Monte Carlo simulations for the nPDF data were carried out using RMCprofile software^35^ combining the atomic movements and swapping and utilizing a 8 × 8 × 9 (77.19 Å × 73.63 Å × 77.21 Å) large supercell containing 30 528 atoms. The XRD and NPD data were analyzed by the Rietveld method, which was carried out using Topas-Academic software^36^ and Jana 2006 software.^37^

Impedance spectroscopy measurements were conducted using a Solartron 1260 frequency response analyzer over the 10^−1^ to 10^7^ Hz frequency range within 300–1000 °C. Impedance data analysis was carried out using ZView software.^38^ The oxygen transport number was determined by electromotive force (EMF) measurements on oxygen concentration cells of O_2_//1%O_2_ and O_2_//5%H_2_, within the temperature range of 500–1000 °C. The theoretical EMF values of the oxygen concentration cells were calculated using the Nernst equation.

Rotation electron diffraction (RED) data were collected on a JEOL-2100 electron microscope operating at 200 kV using a single-tilt tomography sample holder. The RED data were processed using REDp processing software.^39^ X-ray photoelectron spectroscopy (XPS) measurements were performed on an AXIS Supra X-ray photoelectron spectrometer using 200 W monochromated Al Kα radiation. Morphological images and X-ray energy-dispersive spectroscopy (EDS) data were collected on a FEI Tecnai-G2 field emission transmission electron microscope (TEM) equipped with a Li/Si EDX detector. Ultrasoft X-ray absorption spectra (XAS) were collected on the 4B9B beamline of the photoelectron spectroscopy station at the Beijing Synchrotron Radiation Facility (BSRF).

Solid-state ^71^Ga nuclear magnetic resonance (NMR) spectra were acquired on a Bruker AVANCE NEO 850 (20 T) NMR spectrometer with a 1.3 mm magic angle sample spinning probe. All spectra were collected at a rotor frequency of 60 kHz. The 1D quantitative CT-selective ^71^Ga spectra were measured using a Hahn echo experiment with acquisition at the echo top using an interpulse delay of 20 μs. Triple-quantum ^71^Ga MQ-MAS 2D spectra were collected using a three-pulse sequence, a repetition delay of 0.5 s, and rotor-synchronized sampling of the indirect dimension *ν*_r_ = 240 kHz with 128 t_1_ increments. The NMR parameters were fitted by means of the DMfit program.^40^

#### 2.2.1 Atomistic static lattice simulations

Atomistic static lattice simulations on defect formation in SrGa_2_Ge_2_O_8_ were carried out using the General Utility Lattice Program (GULP) on the basis of the interatomic potential approach.^41^ The Buckingham potential function^42^ was used to model interaction between ions, with the shell model to describe the electronic polarizability for the structure modeling of SrGa_2_Ge_2_O_8_. The potential parameters used for the atomistic simulation are listed in Table 1, among which the parameters for La–O, Sr–O, Ga–O, Ge–Ge, and O–O were taken from the previous studies.^22,43^ The potential parameters were slightly modified through the relaxed fitting procedure implemented in the GULP package based on the initial parameters from the study of Tealdi *et al.*,^22^ to better reproduce the crystal structure of SrGa_2_Ge_2_O_8_*via* refining the *A* value while keeping the *C* value fixed. The optimized structural parameters showed good agreement with experimental data (Table S1†), validating the reliability of the interatomic potential model for subsequent static lattice and molecular dynamics simulations. The formation energies of interstitial oxide ions were calculated based on the appropriate combination of dopant, defect, and lattice energies of the binary oxides, as described by the following defect equation: 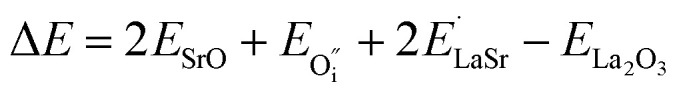.

**Table 1:** Buckingham interatomic potential and shell model parameters for the SrGa_2_Ge_2_O_8_-based materials

Interaction	*A* (eV)	*ρ* (Å)	*C* (eV Å^6^)	*Y* (e)	*k* (eV Å^−2^)
La–O	4579.23	0.30437	0	3	99 999
Sr–O	1400.10150	0.357400	0	1.33	21.53
Ga–O	1241.55690	0.302428	3.323663	0	0
Ge–O	1482.37220	0.324420	10.64850	0	0
O–O	20 784.8710	0.122291	48.06810	−2.869	74.92

#### 2.2.2 Molecular dynamics (MD) simulations

Interstitial oxygen migration in La-doped SrGa_2_Ge_2_O_8_ was investigated using interatomic-potential-based MD simulations performed with the DL_POLY code in the NVT ensemble.^44^ For the undoped composition, a 3 × 3 × 3 supercell model was constructed, comprising 1872 atoms (144 Sr, 288 Ga, 288 Ge, and 1152 O atoms). For the La-doped composition, a simulation box consisting of a 4 × 4 × 4 unit cell was generated, comprising a total of 3360 atoms: 64 La, 192 Sr, 512 Ga, 512 Ge, and 2080 O atoms, corresponding to the composition La_0.15_Sr_0.85_Ga_2_Ge_2_O_8.075_. The systems were equilibrated at 1 atm and various temperatures ranging from 600 to 1200 °C for 135 ps in the *NVT* ensemble (constant number of particles *N*, volume *V*, and temperature *T* controlled using a thermostat). MD data analysis was carried out by using the Visual Molecular Dynamics (VMD) package,^45^ and mean square displacement (MSD) data were exported through the nMoldyn code.^46^ Oxygen diffusion coefficients were determined by linear fitting of the MSD *versus* time curves.

## 3. Results and discussion

### 3.1 Sr_1−*x*_La_*x*_Ga_2_Ge_2_O_8+*x*/2_ solid solution

**Fig. 2:**
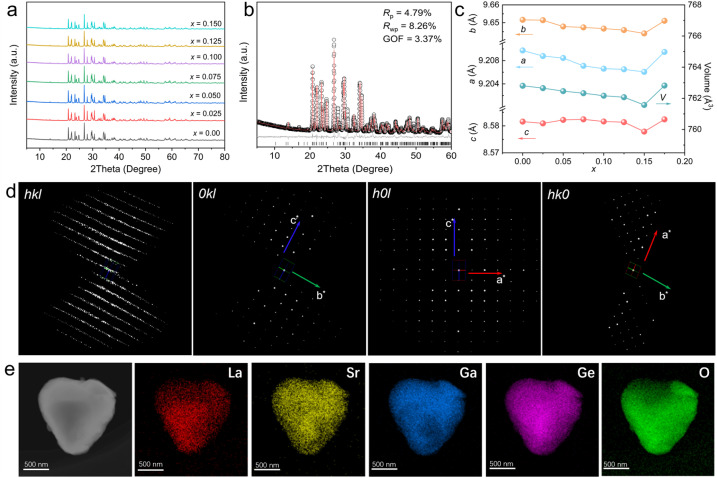
Solid solution of Sr_1-*x*_La_*x*_Ga_2_Ge_2_O_8+*x*/2_. (a) XRD patterns for compositions with 0 ≤ *x* ≤ 0.15. (b) Rietveld refinement plot of Sr_0.85_La_0.15_Ga_2_Ge_2_O_8.075_. (c) Refined cell parameters as a function of *x*. (d) 3D ED reconstructed reciprocal lattice of Sr_0.85_La_0.15_Ga_2_Ge_2_O_8.075_ and the (0*kl*), (*h*0*l*) and (*hk*0) diffraction planes. (e) TEM elemental mapping of Sr_0.85_La_0.15_Ga_2_Ge_2_O_8.075_.

The XRD patterns of La-doped compositions Sr_1−*x*_La_*x*_Ga_2_Ge_2_O_8+*x*/2_ (0 ≤ *x* ≤ 0.15, Fig. 2a) indicate the formation of a pure phase across the entire doping range, with no impurity phases detected. Rietveld refinement of the XRD patterns for the maximum La-doping concentration (*x* = 0.15, Fig. 2b) further confirms the pure phase formation. The refined cell parameters decrease gradually with increasing La content (*x*), attributed to the smaller ionic radius of La^3+^ (1.061 Å) compared to Sr^2+^ (1.13 Å), which causes contraction of the crystal lattice. Three-dimensional continuous rotation electron diffraction (3D cRED) patterns of Sr_0.85_La_0.15_Ga_2_Ge_2_O_8.075_ (Fig. 2d) yield a monoclinic unit cell with space group *P*2_1_/*c* and cell parameters *a* = 9.02 Å, *b* = 9.31 Å, *c* = 8.63 (3) Å, *β* = 90.1°. The observed reflection conditions, including 0*kl*: *k* + *l* = 2*n*, *h*00: *h* = 2*n*; *h*0*l*: *h* + *l* = 2*n*; *h*00: *h* = 2*n*, indicate that the introduction of La^3+^ does not change the average crystal structure of Sr_0.85_La_0.15_Ga_2_Ge_2_O_8.075_. Transmission electron microscopy (TEM) with elemental mapping (Fig. 2e) demonstrates the uniform distribution of all constituent elements in Sr_0.85_La_0.15_Ga_2_Ge_2_O_8.075_, confirming the homogeneous incorporation of La^3+^ into the lattice.

### 3.2 Electrical and stability performances

**Fig. 3:**
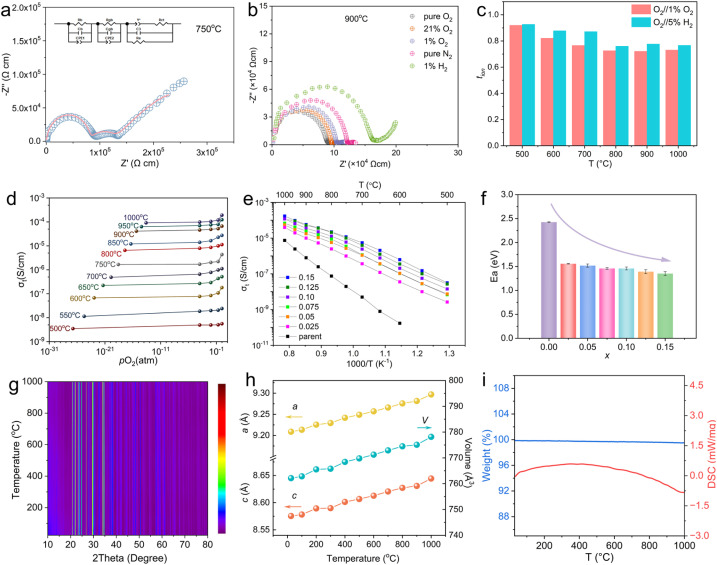
Electrical and stability performance of Sr_1–*x*_La_*x*_Ga_2_Ge_2_O_8+*x*/2_. (a and b) Typical complex impedance plots of *x* = 0.15 at (a) 750 °C in air and (b) 900 °C under different oxygen partial pressures. (c) Oxygen transport numbers of *x* = 0.15 determined using O_2_//1%O_2_ and O_2_//5%H_2_ cells. (d) Conductivities for *x* = 0.15 in an oxygen partial pressure range of 1–10^−30^ atm. (e) Bulk conductivities in air and (f) derived activation energies. (g) VT-XRD patterns of *x* = 0.15 and (h) the refined cell parameters as a function of temperature. (i) TG-DSC curves of *x* = 0.15.

La-doping at the Sr site in SrGa_2_Ge_2_O_8_ can be described using the following defect equation: 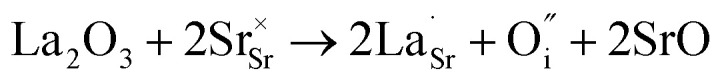. Here, the substitution of La^3+^ for Sr^2+^ introduces interstitial oxygen 
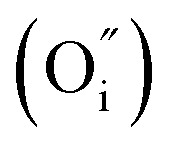
. Alternating current (AC) impedance spectroscopy was used to study the mobility of the introduced interstitial oxygen. The typical complex impedance plots of Sr_0.85_La_0.15_Ga_2_Ge_2_O_8.075_ at different temperatures (Fig. 3a and S1†) show a remarkable Warburg electrode response, indicative of ionic migration. This ionic migration behavior is also observed for the composition with *x* = 0.05 (Fig. S2 and S3†). This electrode response is highly sensitive to oxygen partial pressure, as evidenced in Fig. 3b, further confirming oxide ion migration within the structural framework of Sr_0.85_La_0.15_Ga_2_Ge_2_O_8.075_. The oxygen transport numbers of Sr_0.85_La_0.15_Ga_2_Ge_2_O_8.075_ were determined using an oxygen concentration cell method. Electromotive force measurements were conducted in oxygen concentration cells (O_2_//1%O_2_ and O_2_//5%H_2_, Fig. 3c) over a temperature range of 500–1000 °C, yielding an oxygen transport number of ∼80% with minimal electronic conduction. The conductivities of Sr_0.85_La_0.15_Ga_2_Ge_2_O_8.075_ under different oxygen partial pressures (Fig. 3d) show minor p-type electronic conduction in the high oxygen partial pressure range of 1–10^−4^ atm. The bulk conductivities of Sr_1−*x*_La_*x*_Ga_2_Ge_2_O_8+*x*/2_ (0 ≤ *x* ≤ 0.15, Fig. 3e) show that increasing the concentration of interstitial oxygen enhances oxide ion conductivity by approximately two orders of magnitude compared with the parent composition, while the activation energy decreases from 2.42(1) eV to 1.35(4) eV (Fig. 3f). Among the compositions, the *x* = 0.15 composition, Sr_0.85_La_0.15_Ga_2_Ge_2_O_8.075_, exhibits the highest oxide ion conductivity (1.72 × 10^−4^ S cm^−1^) with an activation energy of 1.35 eV.

Variable temperature XRD (VT-XRD) patterns of Sr_0.85_La_0.15_Ga_2_Ge_2_O_8.075_ (Fig. 3g, and S4†) demonstrate excellent phase stability across the temperature range of 25–1000 °C, further supported by the TG-DSC data (Fig. 3i). Notably, the phase stability is also maintained under reducing conditions (Fig. S5†). The volume thermal coefficient (TEC) value derived from VT-XRD (Fig. 3h) is *α*_V_ = 2.154 × 10^−5^ K^−1^, which closely matches the TEC values of widely used cobalt-containing cathodes (2.0–2.5 × 10^−5^ K^−1^).^47–49^ These thermal stability and compatibility features highlight the potential of the zeolite-like feldspar structure as a promising candidate for SOFC applications.

### 3.3 Average structure

**Fig. 4:**
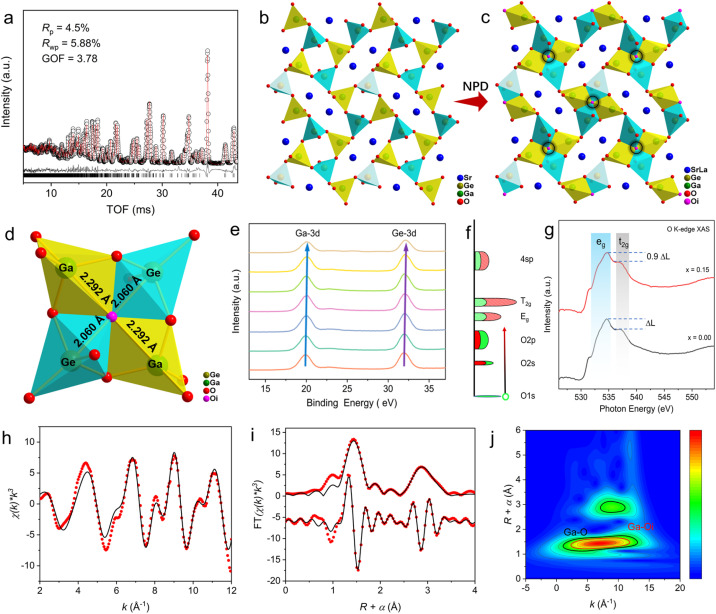
Average structure of Sr_0.85_La_0.15_Ga_2_Ge_2_O_8.075_. (a) Rietveld refinement plot of the NPD data. (b) Parent structure in comparison with (c) crystal structure from NPD data refinement. (d) Enlarged structure around interstitial oxygen in the square ring. (e) Binding energies of Ga-3d and Ge-3d orbitals. (f) Interpretation of the oxygen K-edge XAS spectrum of the 3d transition metal oxide, and the ratio of t_2g_ and e_g_ states is 6 : 4. (g) Ultrasoft O K-edge XAS spectra in comparison with the parent composition. (h) Ga K-edge EXAFS (points) and curve fit (line), shown in *k*^3^ weighted *k*-space. (i) Ga K-edge EXAFS (points) and curve fit (line), shown in *R*-space (FT magnitude and imaginary component). (j) Ga K-edge wavelet transform EXAFS contour plot.

Combining Rietveld analysis with the charge-flipping method applied to the NPD data of Sr_0.85_La_0.15_Ga_2_Ge_2_O_8.075_ (Fig. 4a), using the SrGa_2_Ge_2_O_8_ structural model in monoclinic symmetry (*P*2_1_/*c*) with its Ga and Ge ordering arrangement,^50^ revealed the presence of interstitial oxygen O_i_ at the (0 0 0) site (Table 2). In contrast to the parent structure (Fig. 4b), the interstitial oxygen was located at the center of 4-membered rings rather than the larger 8-membered rings, as marked by black circles in Fig. 4c. The interstitial oxygen, coordinated with two Ga and two Ge atoms, exhibits Ga–O_i_ and Ge-O_i_ bond lengths of 2.292 Å and 2.060 Å, respectively, indicative of underbonding (BVS = 1.30, Table 2) and approximate ionic bonding character (Fig. 4d). This configuration allows the oxygen to remain in a quasi-free state of coordination equilibrium, enabling high mobility. Notably, Ga and Ge adopt (4 + 1) GaO_4+1_ and GeO_4+1_ coordination environments. The introduced interstitial oxygen induces shifts in the binding energies of Ga-3d and Ge-3d orbitals (Fig. 4e) towards higher energy regions, reflecting the influence of the additional oxygen coordination.

**Table 2:** The final refined structural parameters of Sr_0.85_La_0.15_Ga_2_Ge_2_O_8.075_ from NPD data^a^

Atom	Wyckoff site	*x*	*y*	*z*	Occ.	*U*_iso_ (Å^2^)	BVS
Sr1	4*e*	0.8847(5)	0.4135(6)	0.246(1)	0.87(5)	0.040(1)	1.87
La1	4*e*	0.8847(5)	0.4135(6)	0.246(1)	0.13(5)	0.040(1)	2.16
Ga1	4*e*	0.0510(7)	0.1926(8)	0.5691(7)	1	0.033(1)	3.30
Ga2	4*e*	0.2334(7)	0.9240(9)	0.9330(7)	1	0.019(1)	3.65
Ge1	4*e*	0.0555(6)	0.2001(8)	0.9395(6)	1	0.041(1)	3.93
Ge2	4*e*	0.2463(6)	0.9197(9)	0.5567(6)	1	0.025(1)	3.75
O1	4*e*	0.1967(9)	0.091(1)	0.015(1)	1	0.041(1)	2.10
O2	4*e*	0.1908(9)	0.086(1)	0.481(1)	1	0.034(1)	2.19
O3	4*e*	0.120(1)	0.374(1)	0.9337(9)	1	0.031(1)	1.85
O4	4*e*	0.114(1)	0.371(1)	0.5652(9)	1	0.023(4)	1.88
O5	4*e*	0.9110(9)	0.204(1)	1.0760(9)	1	0.040(1)	2.25
O6	4*e*	0.9042(9)	0.193(1)	0.4310(9)	1	0.023(3)	2.21
O7	4*e*	−0.0014(8)	0.1359(6)	0.760(1)	1	0.035(6)	1.75
O8	4*e*	0.332(1)	0.9245(7)	0.743(1)	1	0.031(3)	1.75
O_i_	2*a*	0	0	0	0.10(3)	0.038 (1)	1.30

a Space group: *P*2_1_/*c* (14), *a* = 9.2044(3) Å, *b* = 9.6497(2) Å, *c* = 8.5795 (3) Å, *β* = 90.396(3)°, *V* = 762.01 (4) Å^3^, and *Z* = 4.

To further study the oxygen coordination environments and the associated variations in electronic structure, ultrasoft O K-edge X-ray absorption spectroscopy (XAS) was employed, with Au as the reference (Fig. S6†). The schematic diagram in Fig. 4f illustrates the oxygen K-edge XAS spectrum of the 3d transition metal oxide, with the oxygen 1s core state (shown in green) at ∼534 eV binding energy. Typically, O 1s electrons are excited to occupy the e_g_ and t_2g_ orbitals. However, the presence of excess interstitial oxygen contributes to increased t_2g_ orbital occupancy, as indicated by a reduction in the relative peak intensities from Δ*L* to approximately 0.9Δ*L* (Fig. 4g). As a result, the composition Sr_0.85_La_0.15_Ga_2_Ge_2_O_8.075_ shows a relatively higher t_2g_ orbital occupancy compared to the parent structure, further confirming the impact of the interstitial oxygen in modifying the electrical properties at the atomic scale.

To further investigate the structural variations associated with coordination numbers of B-site Ga, Ga K-edge extended X-ray absorption fine structure (EXAFS) measurements were performed. For the parent SrGa_2_Ge_2_O_8_, the fitting of the Fourier-transformed (FT) *k*^3^-weighted EXAFS spectra in both *k*-space (Fig. S7a†) and *R*-space (Fig. S7b†) was carried out without including the scattering path of the interstitial oxygen, leading to an *R* factor of 0.9% (Table S2†). In contrast, the FT *k*^3^-weighted EXAFS spectra of Sr_0.85_La_0.15_Ga_2_Ge_2_O_8.075_, shown in Fig. 3h and 4i for *k*-space and *R*-space, respectively, were fitted with the inclusion of the scattering path for the interstitial oxygen, giving an *R* factor of 1.7% (Table S3†). Furthermore, wavelet transform analysis of the EXAFS spectra for both the parent SrGa_2_Ge_2_O_8_ (Fig. S7c†) and Sr_0.85_La_0.15_Ga_2_Ge_2_O_8.075_ (Fig. 4j) reveals a weak but discernible signal corresponding to the scattering path of interstitial oxygen. This observation confirms the formation of (4 + 1)-coordinated GaO_4+1_ environments around Ga atoms, induced by the interstitial oxygen, and highlights the role of interstitial oxygen in modulating the local atomic structure.

### 3.4 Local structure

**Fig. 5:**
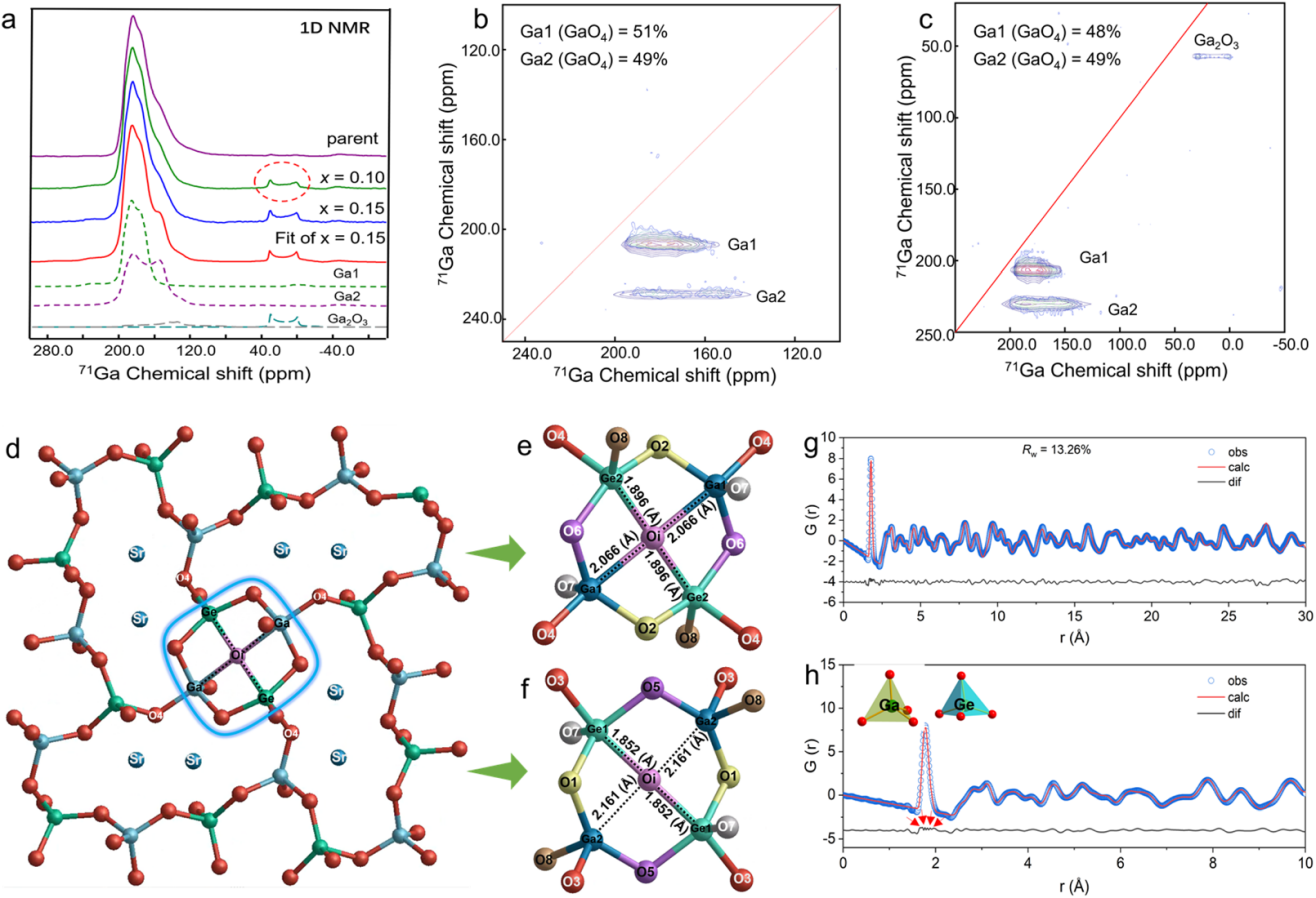
Local structures of Sr_1-*x*_La_*x*_Ga_2_Ge_2_O_8+*x*/2_. (a) ^71^Ga solid-state NMR spectra. (b and c) 2D MQMAS ^71^Ga solid-state NMR spectra of (b) parent SrGa_2_Ge_2_O_8_ and (c) Sr_0.85_La_0.15_Ga_2_Ge_2_O_8.075_. (d) Geometry optimized structure containing interstitial oxygen at (e) Ga1Ge2 and (f) Ga2Ge1 layers. (g) Small box fitting of neutron PDF data of Sr_0.85_La_0.15_Ga_2_Ge_2_O_8.075_ with (h) the short-range fitting in the range of 1–10 Å, where the red arrows indicate mismatches.

^71^Ga solid-state nuclear magnetic resonance (NMR) measurements were performed to study the local structure of Sr_1−*x*_La_*x*_Ga_2_Ge_2_O_8+*x*/2_. The structure of SrGa_2_Ge_2_O_8_ contains two Ga crystallographic sites with a multiplicity ratio of 1 : 1. ^71^Ga MAS NMR spectrum at 20 T and a MAS rate of 60 kHz of pure SrGa_2_Ge_2_O_8_ (Fig. 5a) shows one signal with a complex line shape. In the ^71^Ga MQMAS experiment (Fig. 5b, c and Table 3), two signals were revealed at 197.8 and 204.0 ppm. The simulation of the 1D ^71^Ga spectrum yielded an integral intensity ratio of 1 : 1, in good agreement with the crystal structure. Compared with the parent composition, the La-doped compositions displayed essentially the same ^71^Ga resonance signals except for one additional quadrupolar signal at 40.7 ppm with a *C*_Q_ value of 8.4 MHz and an *η*_Q_ value of 0 (Fig. 5a and c). Based on literature data we assigned this resonance to six-fold coordinated gallium in gallium oxide.^51,52^ Ga_2_O_3_ contains two Ga crystallographic sites with a multiplicity ratio of 1 : 1. The second signal from the four-coordinated gallium of Ga_2_O_3_ oxide was not observed due to its low-intensity, second-order quadrupolar enlargement, and the overlap of the intense SrGa_2_Ge_2_O_8_ signals. It should be noted that the spectrum of parent SrGa_2_Ge_2_O_8_ contains the trace of this signal from the 6-coordinate Ga. The existence of Ga_2_O_3_ in the samples was not detected by XRD, indicating the higher sensitivity of NMR to traces of secondary phases.

**Table 3:** ^71^Ga isotropic chemical shifts (*δ*_iso_), quadrupolar constants (*C*_Q_), asymmetry parameters (*η*_Q_), and integral intensities (*I*) obtained from the simulation of the ^71^Ga MAS and MQMAS spectra at 20 T of Sr_1−*x*_La_*x*_Ga_2_Ge_2_O_8+*x*/2_

Atom	*δ*_iso_, ppm (±0.2 ppm)	*C*_Q_, MHz (±0.1 MHz)	*η*_Q_ (±0.05)
Ga1	197.8	6.7	0.39
Ga2	204.0	9.8	0.27
Ga_2_O_3_	40.7	8.4	0
	200.0	11.0	0.85

The nearly unchanged ^71^Ga resonance signals in the NMR spectra of the La-doped compositions compared with the parent one suggest that locally the interstitial oxide ions do not form strong bonding with Ga. This is consistent with the NPD structural analysis (Fig. 4d), which revealed that Ga forms 4 covalent bonds (1.828 Å) with lattice oxygen and one ionic bond (2.292 Å) with interstitial oxygen. The coordination of gallium close to oxygen interstitials in La-doped compositions can thus be described as 4 + 1. Unfortunately, for this reason, probing structural variations arising from interstitial oxygen in the ^71^Ga NMR spectra could be hindered given the low concentration of interstitial oxide ions and the much weaker ionic bonding nature of Ga–O_i_. Our conclusion is confirmed by observations in the case of La_2_Ga_3_O_7.5_ in which the Ga1 site has 4 + 1 coordination with a chemical shift (*δ*_iso_ = 187.0 ppm) in the range corresponding to four-coordinated gallium.^53^

Geometry optimization of the Sr_0.85_La_0.15_Ga_2_Ge_2_O_8.075_ structure revealed structural features consistent with the average structure (Fig. 5d–f) and yielded a defect formation energy of ∼1.80 eV, indicating a thermodynamically favorable environment for accommodating interstitial defects within the zeolite-like feldspar structure. Although the defect formation energy is significantly lower than that (2.67 eV) observed for the typical melilite La_1.50_Sr_0.50_Ga_3_O_7.25_, this feldspar phase has a much lower capability for accommodating interstitial oxide ion defects than the melilite. Therefore, the defect formation energies cannot be directly compared to quantitatively assess the defect formation capability particularly when derived from calculations based on interatomic potential methods using empirical parameters. The interstitial defect content in the feldspar phase could be experimentally modulated further using different synthetic routes, as indicated by the melilite gallate and aluminates.^53,54^

The interstitial oxygen in the average structure is in a quasi-free state of coordination equilibrium, which presumably could promote its high mobility. However, this contrasts with the experimentally observed limited interstitial oxygen mobility in Sr_0.85_La_0.15_Ga_2_Ge_2_O_8.075,_ which is closely related to local structural variations that are not captured in the average structure. Although spectroscopic techniques such as O-XAS, ^71^Ga-EXAFS, and solid-state NMR revealed some information on the local structure, to gain deeper insights into the local structures, the neutron partial distribution function (nPDF) technique was employed on Sr_0.85_La_0.15_Ga_2_Ge_2_O_8.075_. Indeed, small-box fitting of the nPDF data for Sr_0.85_La_0.15_Ga_2_Ge_2_O_8.075_ (Fig. 5g) and short-range fitting within the range of 0–10 Å (Fig. 5h) revealed mismatches, indicated by red arrows in Fig. 5h. These mismatches indicate the presence of local structural disorder/distortions in Sr_0.85_La_0.15_Ga_2_Ge_2_O_8.075_, which could have correlation with the interstitial oxygen migration behavior. These local structural disorder/distortions were further investigated through reverse Monte Carlo (RMC) simulations using large supercell models fitted to the PDF data.

### 3.5 Collective local distortion and correlated disorder in Sr_1−*x*_La_*x*_Ga_2_Ge_2_O_8+*x*/2_

**Fig. 6:**
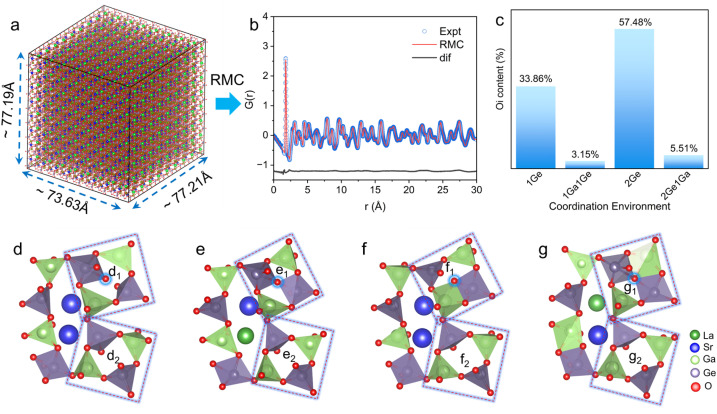
Local structure of Sr_0.85_La_0.15_Ga_2_Ge_2_O_8.075_ revealed by RMC simulations on neutron PDF data. (a) 3D atomic configuration with an approximately 77.19 Å × 73.63 Å × 77.21 Å cell for RMC modeling. (b) Big box refinement of neutron PDF data. (c) The proportion of interstitial oxygen in different coordination environments from the statistics of the RMC-fitted model. (d–f) Coordination environments of interstitial oxygen: coordinated with (d) one Ge, (e) two Ge, (f) one Ga and one Ge, and (g) one Ga and two Ge.

To further elucidate the local structure and its correlation with interstitial oxygen migration behavior in Sr_0.85_La_0.15_Ga_2_Ge_2_O_8.075_, a large-scale big-box model was constructed using an 8 × 8 × 9 supercell (77.19 Å × 73.63 Å × 77.21 Å, Fig. 6a). The reverse Monte Carlo (RMC) method was used to analyse the spatial variation of local atomic distributions in Sr_0.85_La_0.15_Ga_2_Ge_2_O_8.075_. The big box refinement of neutron PDF data of Sr_0.85_La_0.15_Ga_2_Ge_2_O_8.075_ (Fig. 6b) reveals various local structures (Fig. 6c–g) responsible for stabilizing interstitial oxygen. By tracking interstitial oxygens, we employed statistical analysis of the RMC-derived big-box model to elucidate the stabilization mechanism of interstitial oxygens. The statistical analysis reveals that all interstitial oxygens are stabilized through collective tetrahedral distortions (Fig. 6d_2_–g_2_) that facilitate coordination with Ge atoms (Fig. 6c and d_1_–g_1_), revealing the correlated disorder of interstitial oxygen coordinated with Ge. Specifically, these interstitial oxygens predominantly coordinate with one Ge atom (31.86%, Fig. 6d_1_) or two Ge atoms (57.48%, Fig. 6e_1_), with additional configurations involving one Ga and one Ge atom (3.15%, Fig. 6f_1_) or two Ge atoms and one Ga atom (5.51%, Fig. 6g_1_). In other words, the local collective tetrahedral distortions arise from the preferential coordination of interstitial oxygen with Ge atoms, accompanied by cooperative local relaxations of the surrounding atoms to accommodate the interstitial oxygen and maintain its coordination with Ge.

This behavior arises from the differences in electronegativity (*χ*) and ionic radius (*r*) between Ge (*χ* = 2.01, *r* = 0.39 Å) and Ga (*χ* = 1.81, *r* = 0.47 Å). The higher electronegativity and smaller ionic radius of Ge favor stronger covalent bonding with interstitial oxygen, inducing a confinement effect that traps interstitial oxygens within the 4-membered rings of the zeolite-like feldspar framework. This similar preferential coordination of interstitial oxygen with Ge is also observed in the B-site mixed melilite structure.^55^ In other words, while local collective distortions facilitate the accommodation of interstitial oxygens, they simultaneously impose geometric constraints that limit long-range ion transport. These findings provide direct evidence of the structural variations induced by the incorporation of interstitial oxygen and its role in modulating local ionic mobility: the mobility of the interstitial oxygen ions is stabilized and facilitated by local collective distortions within the GaO_4_ and GeO_4_ tetrahedra, but is trapped by coordination with Ge atoms within 4-membered rings. This disrupts the coordination equilibrium quasi-free state of interstitial oxygen determined in the average structure, highlighting the crucial role of local structural variations in correlation with the experimentally observed low oxide ion mobility in Sr_0.85_La_0.15_Ga_2_Ge_2_O_8.075_.

### 3.6 Interstitial oxygen migration mechanism

**Fig. 7:**
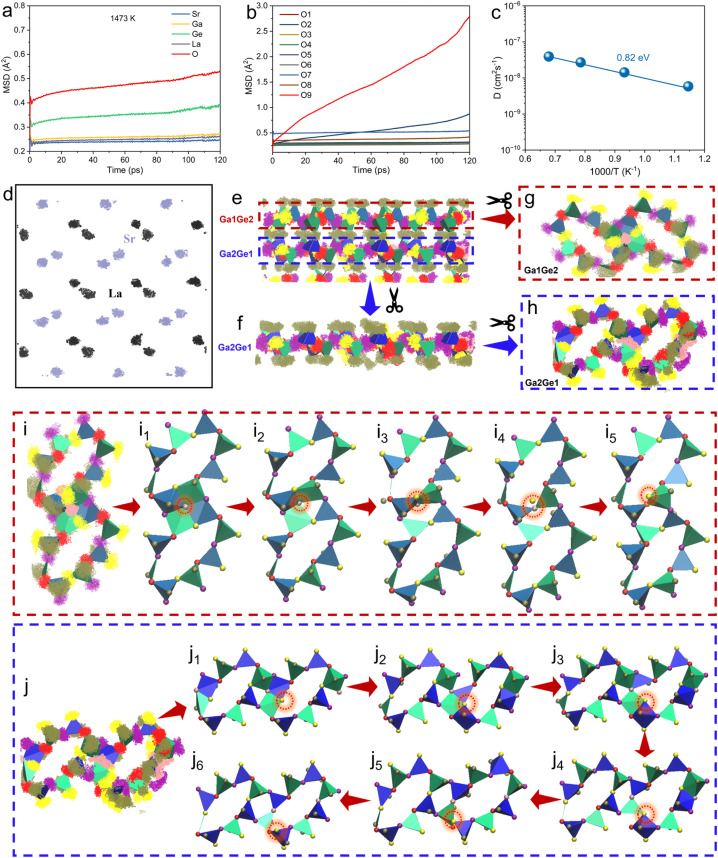
Interstitial oxygen migration in Sr_0.85_La_0.15_Ga_2_Ge_2_O_8_. (a) Mean square displacement (MSD) values of Sr, La, Ga, Ge, and O atoms. (b) MSD values for oxygen atoms at different lattice sites. (c) Arrhenius plot of oxide ion diffusion coefficients. (d) Scattering plots of La and Sr cations. (e) Scattering plots viewed along the [010] direction. (f and h) Scattering plots of the Ga2Ge1 layer and (g) the Ga1Ge2 layer, extracted from (e). (i) Oxide ion migration pathways in the Ga1Ge2 layer and (j) Ga2Ge1 layer. Deep blue and light blue tetrahedra represent Ga1O_4_ and Ga2O_4_ tetrahedra, respectively; deep green and light green tetrahedra correspond to Ge2O_4_ and Ge1O_4_ tetrahedra. Oxygen atoms are color-coded as follows: red/yellow (O3/O4), purple (O1/O2), brown (O5/O6), pink (O7/O8), and orange (O9).

To explore the underlying mechanism of interstitial oxygen transport in Sr_0.85_La_0.15_Ga_2_Ge_2_O_8.075_, molecular dynamics (MD) simulations based on the interatomic potential method were employed. The mean square displacement (MSD) values for cations and oxide ions in the parent SrGa_2_Ge_2_O_8_ (Fig. S8†) show only lattice vibrations within the temperature range of 873–1473 K, with no evidence of long-range ionic migration. In contrast, MSD analysis for Sr_0.85_La_0.15_Ga_2_Ge_2_O_8.075_ (Fig. 7a and b) reveals remarkable long-range migration of interstitial oxygens, accompanied by localized but non-negligible motions of Ge cations, while all other cations exhibit only lattice vibrations. These small but non-negligible motions of Ge atoms (Fig. 7a) indicate that effective interstitial ion transport likely requires local motions of Ge atoms, coupled to the dynamics of the surrounding Ge/GaO_4_ frameworks. Notably, the interstitial oxygens exhibit long-range migration with an activation energy of 0.82 eV and oxide ion diffusion coefficients ranging from 5.8 × 10^−9^ to 3.9 × 10^−8^ cm^2^ s^−1^ over the temperature range of 873–1473 K (Fig. 7c), highlighting the significant enhancement in oxide ion mobility introduced by the La doping.

The scatter plots of cationic and oxide ion positions for Sr_0.85_La_0.15_Ga_2_Ge_2_O_8.075_ (Fig. 7d and e) reveal that all oxygen atoms are involved in oxygen exchange without any cationic exchange, consistent with the above MSD results. To analyse the oxide ion migration pathways in more detail, the scatter plots for the Ga1Ge2 layer and Ga2Ge1 layer were extracted and are shown in Fig. 7g and h, respectively. In the Ga1Ge2 layer (Fig. 7i), it is observed that the interstitial oxygen is initially stabilized within the 4-membered ring formed by two GaO_4_ and two GeO_4_ tetrahedra (Fig. 7i_1_). During the simulation, the interstitial oxygen transitions between coordination states associated with its migration. The interstitial oxygen then coordinates with two GaO_4_ and one GeO_4_ tetrahedra (Fig. 7i_2_), followed by coordination with one GaO_4_ and one GeO_4_ tetrahedra (Fig. 7i_3–4_). Eventually, the interstitial oxygen coordinates with a single GaO_4_ tetrahedron within a larger 8-membered ring (Fig. 7i_5_), realizing the long-range migration of interstitial oxide ion from the 4-membered ring to the 8-membered ring in the zeolite-like structure. This migration is driven by local structural distortions in the GaO_4_ and GeO_4_ tetrahedra, which create dynamic pathways for oxide ion migration. The local structural distortions in the GaO_4_ and GeO_4_ tetrahedra disrupt the local lattice symmetry and facilitate oxide ion mobility. The same migration mechanism is also observed in the Ga2Ge1 layer (Fig. 7j and j_1–6_) in Sr_0.85_La_0.15_Ga_2_Ge_2_O_8.075_. These MD simulation results are consistent with the local structural variations derived from RMC modeling.

The radial distribution functions (RDFs) of Ga/Ge–O interactions (Fig. S9–S16†), derived from MD simulations, provide valuable insight into the local structural disorder in Sr_0.85_La_0.15_Ga_2_Ge_2_O_8.075_. In the RDFs of Ga2–O interactions over the simulation time range of 0–120 ps, it is observed that the originally coordinated oxygen atoms (O1, O3, O5, and O8, Fig. S9†) surrounding Ga2, are gradually joined by additional oxygen atoms (O2, O6, and O9, Fig. S9†). This behavior is also evident in other Ga–O and Ge–O (Fig. S10–S16†) interactions, demonstrating that all oxygen sites participate in the long-range oxide ion migration.

Zeolite and zeolite-like feldspar-type frameworks can be transformed into oxide-ion conductors through targeted local structural modifications. The targeted aliovalent La^3+^ → Sr^2+^ substitution employed here is one example of such a strategy, readily extendable to other open-framework systems, including aluminogermanates, aluminophosphates, and gallium phosphates. In addition, the selection of B-site framework cations with larger ionic radii and variable coordination numbers (*e.g.*, Ga or Ge instead of Al) can expand tunnel dimensions and promote coordination flexibility, both of which are critical for enhancing ionic mobility. In contrast, Si-based frameworks may be less suitable due to the rigid tetrahedral coordination preference of Si, which limits both structural flexibility and ionic mobility.^56–58^ We further propose that zeolite-like frameworks incorporating compositionally pure B-site cations capable of variable coordination represent a promising and underexplored class of oxide-ion conductors. Taken together, these insights establish that oxide-ion transport in zeolite-like structures is governed by the interplay between framework openness, B-site coordination flexibility, and aliovalent doping, providing a promising space for discovering related materials across the broader zeolite family.

The findings highlight the exceptional potential of the zeolite-like feldspar structure for developing interstitial oxide ion conductors for SOFCs, extending its utility beyond traditional applications in building materials and dielectric ceramics.^59^ Although the interstitial oxygen in zeolite-like case Sr_0.85_La_0.15_Ga_2_Ge_2_O_8.075_ exhibits limited mobility due to higher migration energies induced by the correlated disorder of interstitial oxygen coordinationed with Ge, its favorable ability to accommodate of interstitial oxygen defects suggests promising applications in catalytic processes where ion mobility is less critical. Moreover, zeolite-like structures featuring interstitial oxygen defects offer additional advantages, including increased active sites, tunable electronic structures to enhance catalytic activity, optimized redox cycles, and improved reaction selectivity. These characteristics render zeolite-like structures attractive for applications in electrocatalysis, water splitting, CO_2_ reduction, methane oxidation, and exhaust treatment. Furthermore, in many cases, the information derived from the averaged structure often masks critical local structural details, making it challenging to correlate structural features with the ionic transport behaviors in materials. Therefore, employing research methods that focus on local structural investigations has become increasingly essential. In this study, NMR, EXAFS, and neutron PDF based on RMC modeling offer the advantages of uncovering structural features hidden within the average structure. Notably, we identify the correlated disorder of interstitial oxygen species coordinated with Ge atoms within 4-membered rings, accompanied by local collective distortions of neighboring GaO_4_ and GeO_4_ tetrahedra, which act as carrier traps during oxide ion migration. These features are distinct from both uncorrelated simple local distortions, referring to isolated displacements of individual atoms or small clusters from their ideal lattice positions, and conventional periodic supercell modulations.^60,61^ By bridging the gap between local structures and ionic migration behavior, this study uncovers fundamental mechanisms hidden within the average structure and offers a pathway for designing and optimizing ion-conducting materials through tailored modifications of local structures in zeolite-like frameworks.

## 4. Conclusions

In summary, the zeolite-like structural family, exemplified by strontium feldspar Sr_1−*x*_La_*x*_Ga_2_Ge_2_O_8+*x*/2_, has been demonstrated as a new structure type for accommodating and transporting interstitial oxide ions. NPD analysis revealed that interstitial oxygen is located at the center of 4-membered rings, coordinated to two Ga and two Ge atoms in a coordination equilibrium quasi-free state, presumably with high mobility within the average structural framework, which however contrasts with the experimentally observed low mobility. Multiple complementary local structural analysis techniques, including 1D and 2D ^71^Ga NMR, EXAFS, and neutron PDF based on RMC modeling, demonstrated that the stabilization of interstitial oxygen is facilitated by local collective distortions within the GaO_4_ and GeO_4_ tetrahedra, but its migration is hindered by the correlated disorder of interstitial oxygen coordinated with Ge atoms within 4-membered rings. The resulting material, Sr_0.85_La_0.15_Ga_2_Ge_2_O_8.075_, demonstrates excellent thermo-mechanical stability and a suitable TEC value well-matched to that of widely used cobalt-containing cathodes. Although its moderate oxide-ion conductivity and non-negligible electronic conductivity constrain its applicability as a SOFC electrolyte, the combination of a zeolite-like framework capable of accommodating oxygen defects and high thermal stability and compatibility indicates that the zeolite-like family holds strong potential not only for the development of advanced oxide-ion conductors, if the oxide ion mobility can be enhanced, but also for the catalytic applications associated with breaking and reforming of metal–oxygen bonds assisted by and correlated with oxide ion mobility. This finding not only demonstrates the zeolite-like feldspar structure as a new family of oxide ion conductors but also bridges the gap between local structural variations and ionic migration behavior, providing a solid foundation for designing and optimizing ion-conducting materials through local structural modifications in zeolite-like frameworks.

## Author contributions

X. K. and X. L. supervised the experimental design and led the overall study. X. W. performed the majority of the experiments as well as data analysis. A. R. assisted with solid-state NMR measurements and contributed to the discussion and interpretation of NMR data. C. L. helped with the neutron PDF data collection. S. D. and L. H. help with the NPD data collection. C. L., H. X., K. L., Q. L., J. S., X. X., participated in the careful discussion on the local structure of zeolite-like structures. The corresponding authors of this manuscript are X. K. and X. L. All authors discussed the results and commented on the manuscript.

## Conflicts of interest

The authors declare no competing interests.

## Supplementary Material

SC-OLF-D5SC02898A-s001

## Data Availability

The authors confirm that the data supporting the findings of this study are available within the article and its ESI.†
