# Supplementary material for: Unlocking zeolite-like structures as a new family of interstitial oxide ion conductors: insights into carrier trapping, collective local distortion, and correlated disorder
Source: Chem Sci. 2025 Jul 16;16(33):15141–54. doi: 10.1039/d5sc02898a (PMC12282666; doi:10.1039/d5sc02898a)
Supplement: SC-016-D5SC02898A-s001 [file SC-016-D5SC02898A-s001.pdf]

Supplementary Information (SI) for Chemical Science.
This journal is © The Royal Society of Chemistry 2025

Supporting Information for:

Unlocking Zeolite-Like structure as a New Family of Interstitial Oxide Ion Conductors: Insights into Carrier Trapping, Collective Local Distortion, and Correlated Disorder

Xianyi Wei,[a] Xiaohui Li,[a*] Aydar Rakhmatullin,[b] Xiaoge Wang,[c] Cheng Li,[d] Hankun Xu,[e] Sihao Deng,[f] Lunhua He,[f,g] Kun Lin,[e] Qiang Li,[e] Junliang Sun,[c] Xianran Xing,[e] and Xiaojun Kuang*[a]

[a] Guangxi Key Laboratory of Electrochemical and Magnetochemical Functional Materials, College of Chemistry and Bioengineering, Guilin University of Technology, Guilin 541004, People's Republic of China.

[b] Conditions Extremes et Materiaux: Haute Temperature et Irradiation (CEMHTI-CNRS), CEMHTI UPR3079, University of Orléans, F-45071 Orléans, France.

[c] College of Chemistry and Molecular Engineering, Peking University, Beijing National Laboratory for Molecular Science (BNLMS), Beijing 100871, People's Republic of China.

[d] Oak Ridge National Laboratory, Neutron Sciences Directorate, Oak Ridge, TN, 37831-2008, United States of America.

[e] Beijing Advanced Innovation Center for Materials Genome Engineering, Institute of Solid State Chemistry, University of Science and Technology Beijing, Beijing 100083, People's Republic of China.

[f] Songshan Lake Materials Laboratory, Dongguan 523808, China; Spallation Neutron Source Science Center, Dongguan 523803, People's Republic of China.

[g] Beijing National Laboratory for Condensed Matter Physics, Institute of Physics, Chinese Academic of Sciences, Beijing 100190, People's Republic of China.

E-mail address: xiaohuili@glut.edu.cn.; kuangxj@glut.edu.cn.

Table S1. Calculated and experimental structural parameters for $SrGa_2Ge_2O_8$

Parameters	Calculated	Experimental	Δ(Calc.-Exp.)
a (Å)	9.2925	9.2100	0.0825
b (Å)	9.5857	9.6660	-0.0803
c (Å)	8.6515	8.5700	0.0815
$\alpha = \gamma$ (°)	90	90	0
β (°)	90.5154	90.5600	-0.446
Volume (Å^3)	770.6112	762.8980	7.7132
Sr1-O1 (Å)	2.9335	2.6472	0.2863
Sr1-O2 (Å)	2.9847	2.7056	0.2791
Sr1-O3 (Å)	3.3077	3.4740	-0.1663
Sr1-O3 (Å)	2.7673	2.6244	0.1429
Sr1-O4 (Å)	3.3357	3.4754	-0.1397
Sr1-O4 (Å)	2.8066	2.5893	0.2173
Sr1-O5 (Å)	2.7338	2.5807	0.1531
Sr1-O6 (Å)	2.7100	2.6016	0.1084
Sr1-O8 (Å)	2.8891	2.6219	0.2672
Ga1-O2 (Å)	1.6806	1.8406	-0.16
Ga1-O4 (Å)	1.6841	1.8367	-0.152
Ga1-O6 (Å)	1.6743	1.8290	-0.1547
Ga1-O7 (Å)	1.6395	1.8040	-0.1645
Ga2-O1 (Å)	1.6633	1.8209	-0.1576
Ga2-O3 (Å)	1.6825	1.8436	-0.1611
Ga2-O5 (Å)	1.6659	1.8140	-0.1481
Ga2-O8 (Å)	1.6620	1.8415	-0.1795
Ge1-O1 (Å)	1.7356	1.7566	-0.021
Ge1-O3 (Å)	1.7501	1.7481	0.002
Ge1-O5 (Å)	1.7346	1.7487	-0.0141
Ge1-O7 (Å)	1.7006	1.7343	-0.0337
Ge2-O2 (Å)	1.7205	1.7412	-0.0207
Ge2-O4 (Å)	1.7501	1.7652	-0.0151
Ge2-O6 (Å)	1.7240	1.7390	-0.015
Ge2-O8 (Å)	1.7308	1.7503	-0.0195

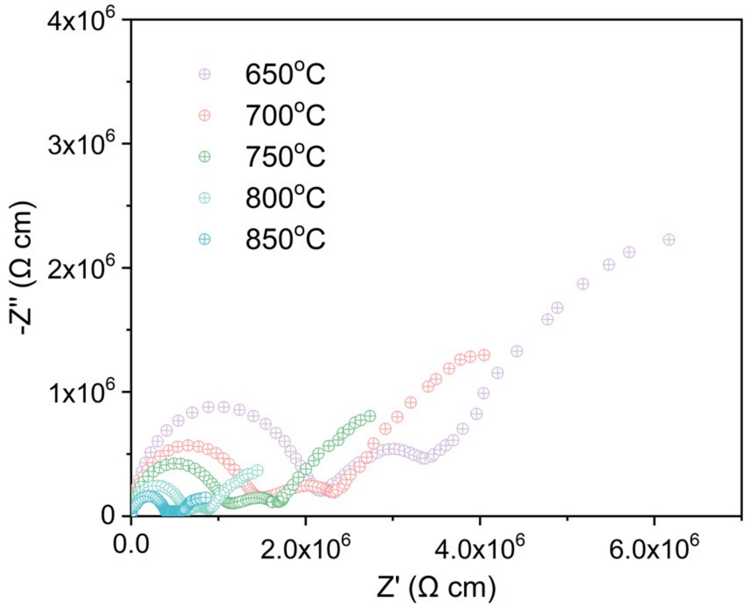

Figure S1. Complex impedance plots of $x = 0.15$ composition at different temperatures.

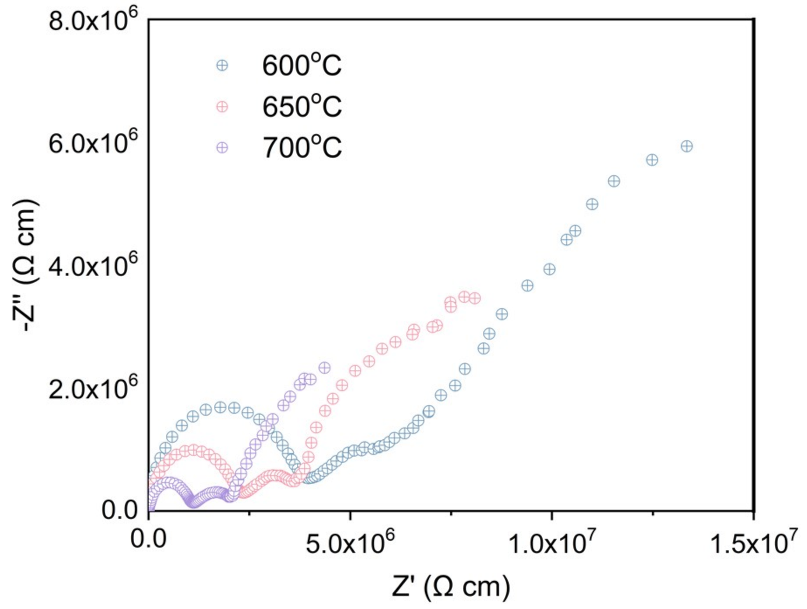

Figure S2. Complex impedance plots of $x = 0.05$ composition at different temperatures (600 °C, 650 °C, and 700 °C).

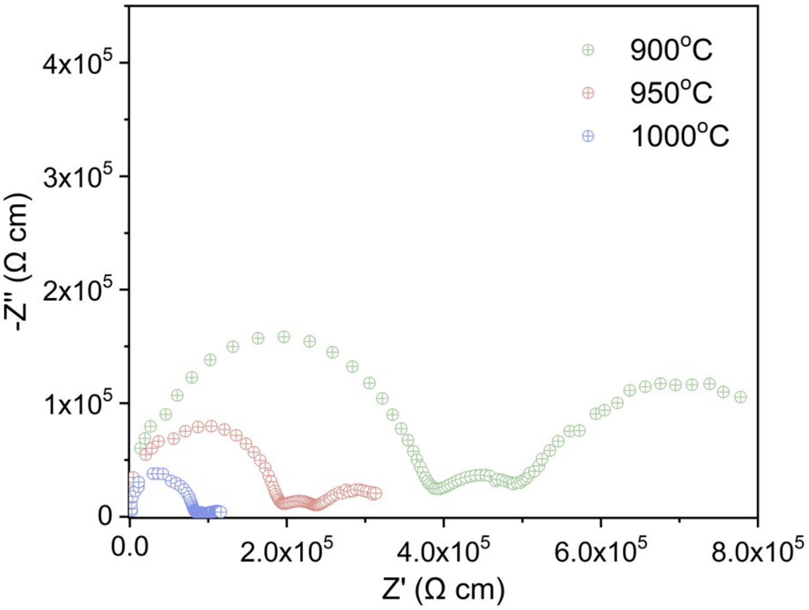

Figure S3. Complex impedance plots of $x = 0.05$ composition at different temperatures (900 °C, 950 °C, and 1000 °C).

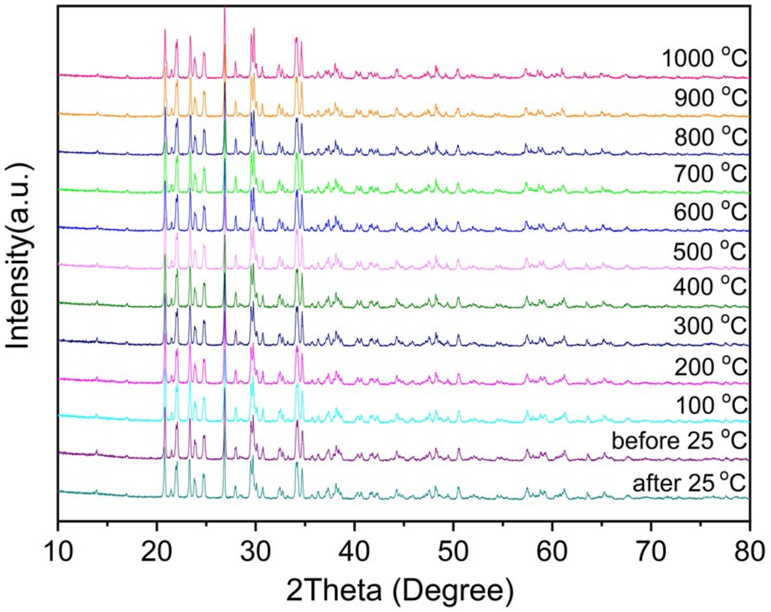

Figure S4. Variable temperature XRD patterns of $Sr_{0.85}La_{0.15}Ga_2Ge_2O_{8.075}$.

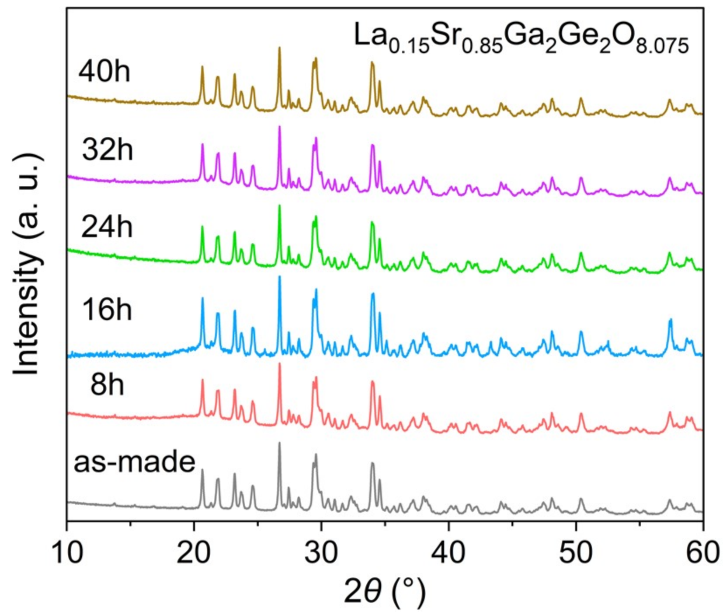

Figure S5. XRD patterns of $Sr_{0.85}La_{0.15}Ga_2Ge_2O_{8.075}$ reduced at 800°C for 8-40 h under a 5% H_2-95% N_2 atmosphere.

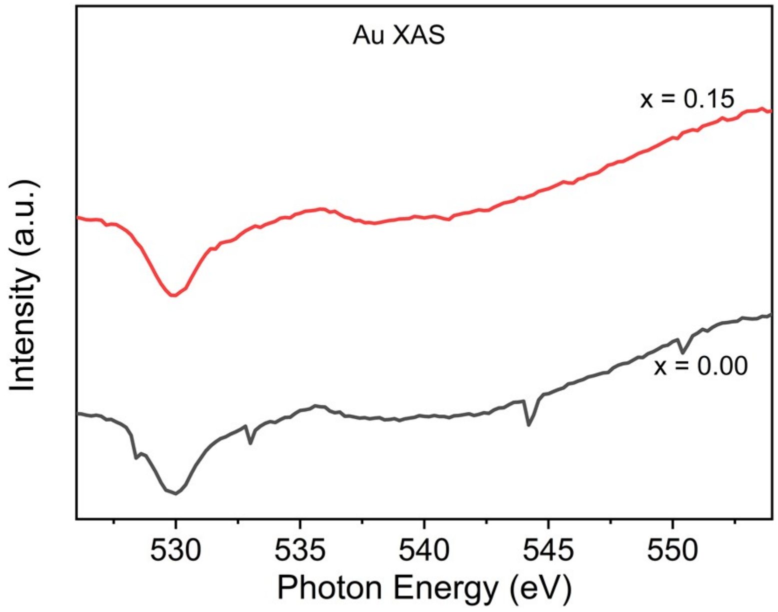

Figure S6. XAS spectra of Au as a reference to $Sr_{1-x}La_xGa_2Ge_2O_{8+x/2}$.

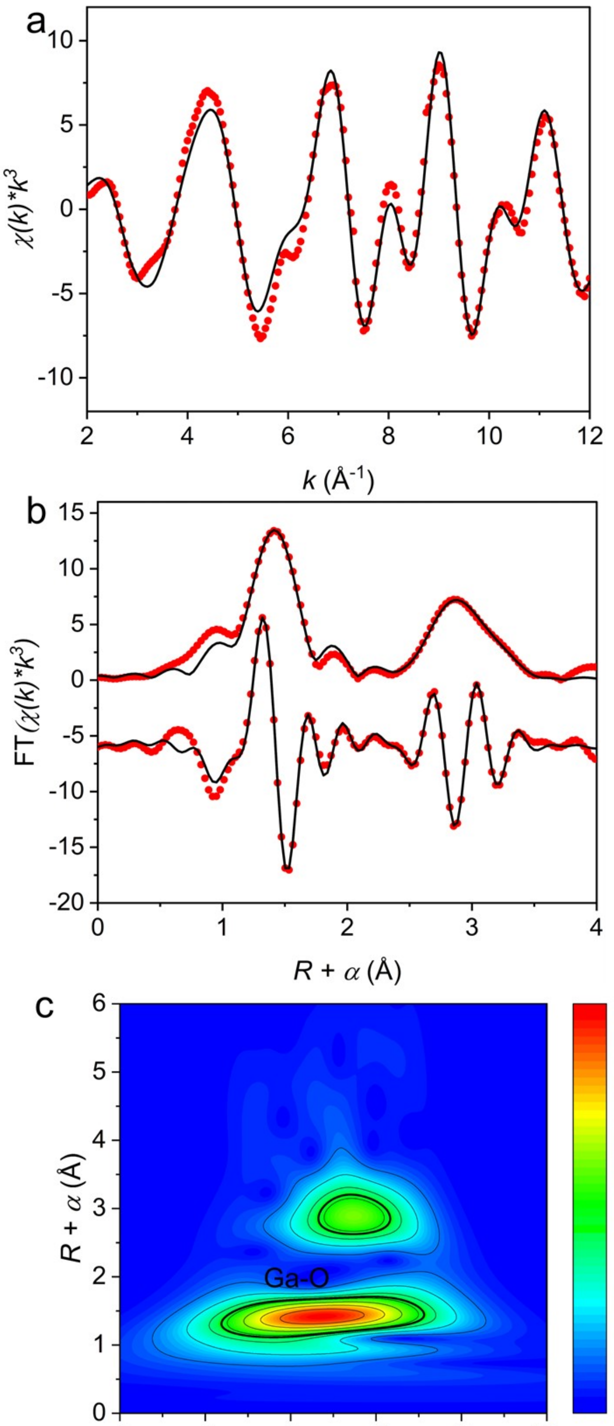

Figure S7. Fourier-transformed (FT) k3-weighted EXAFS (points) and curvefit (line) spectra in both (a) *k*-space and (b) *R*-space for parent $SrGa_2Ge_2O_8$. (c) Ga K-edge wavelet transform EXAFS contours of parent $SrGa_2Ge_2O_8$.

Table S2. Curvefit Parameters for Ga K-edge EXAFS for $SrGa_2Ge_2O_8$

Path	*N*	*R* / Å	*d* / Å	σ^2 / Å^2
O5	4.0	1.83(1)	1.830	0.001
Sr1	1.0	3.46(1)	3.422	0.001
Ge1	3.0	3.13(1)	3.141	0.004
Ge2	1.0	2.51(1)	3.175	0.018
O1	18.0	3.64(1)	3.307	0.073

* S_0^2 was fixed as 0.776. $\triangle E_0$ was refined as a global fit parameter, returning a value of (3±1) eV. Data ranges: $2 \leq k \leq 12$ Å^{-1}, $1 \leq R \leq 3$ Å. The number of variable parameters is 12, out of a total of 15.7 independent data points. *R* factor for this fit is 0.9%.

Table S3. Curvefit Parameters for Ga K-edge EXAFS for $La_{0.15}Sr_{0.85}Ga_2Ge_2O_{8.075}$

Path	*N*	*R* / Å	*d* / Å	σ^2 / Å^2
O5	4.0	1.84(1)	1.730	0.001
Oi	1.0	2.42(1)	2.292	0.006
Sr1	1.0	2.92(1)	2.994	0.002
Ge1	3.0	3.13(1)	3.081	0.001
Ge2	1.0	3.54(1)	3.244	0.001
O5	18.0	3.25(1)	3.249	0.006

* S_0^2 was fixed as 0.776. $\triangle E_0$ was refined as a global fit parameter, returning a value of (7±2) eV. Data ranges: $2 \leq k \leq 12$ Å^{-1}, $1 \leq R \leq 3$ Å. The number of variable parameters is 12, out of a total of 15.7 independent data points. R factor for this fit is 1.7%.

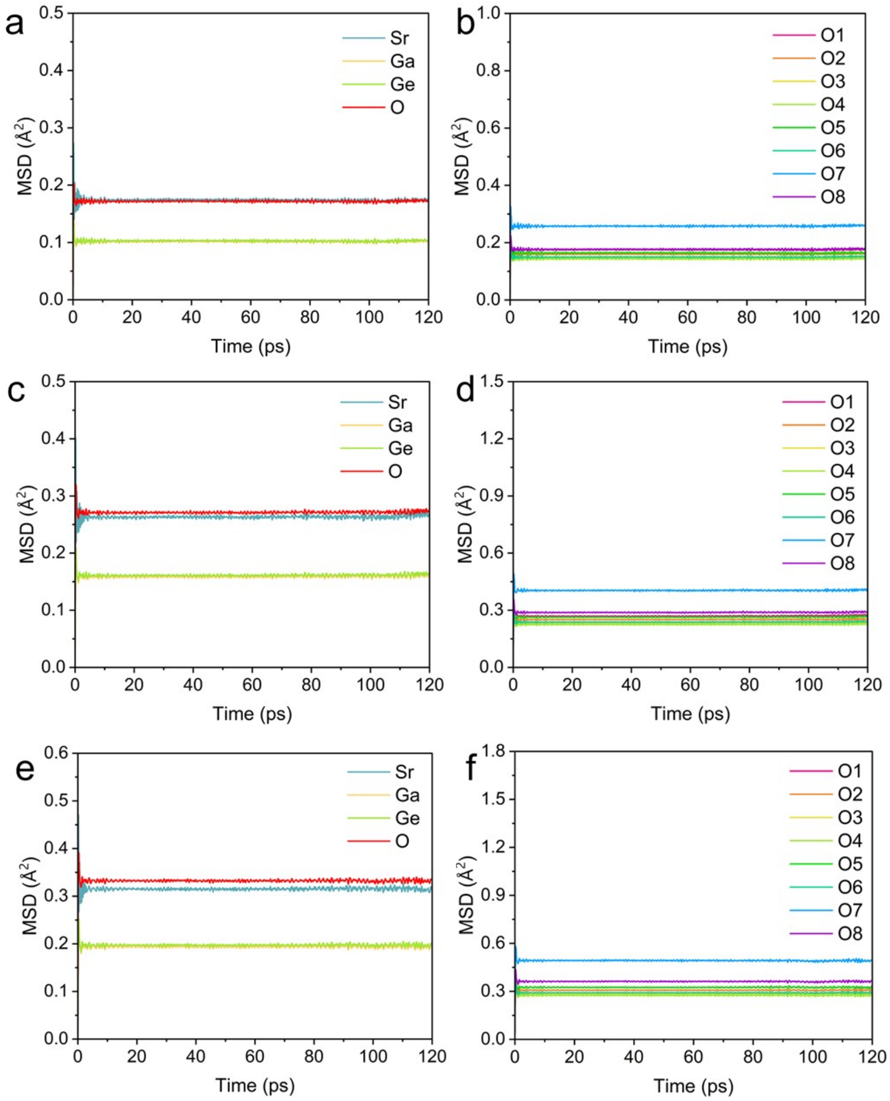

Figure S8. MSD values of Sr, Ga, Ge, and O in parent $SrGa_2Ge_2O_8$ at (a-b) 873 K, (c-d) 1273 K, and (e-f) 1473 K.

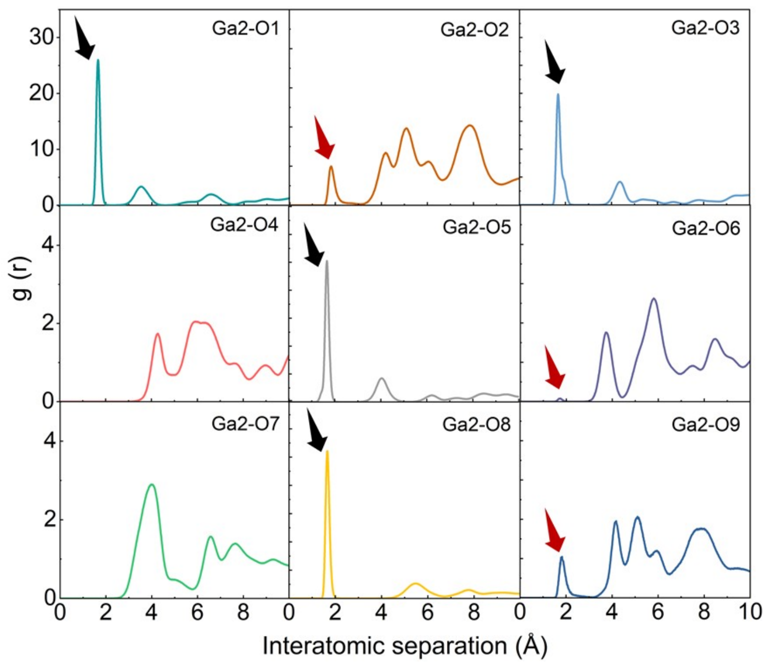

Figure S9. RDFs of Ga2-O interactions with simulation times, where the black and red arrows denote the interactions of Ga2 with the original site O (O1, O3, O5 and O8) and the derivative site O (O2, O6, and O9), respectively.

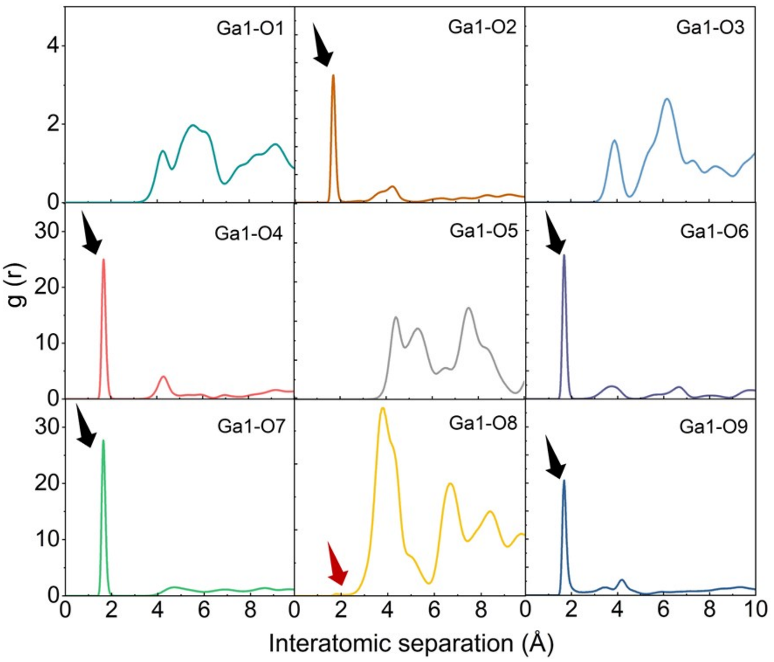

Figure S10. RDFs of Ga1-O interactions with the simulation time within the Ga1Ge2 layer, where the black arrows denote the interactions of Ga1 with the original site O (O2, O4, O6, O7 and O9).

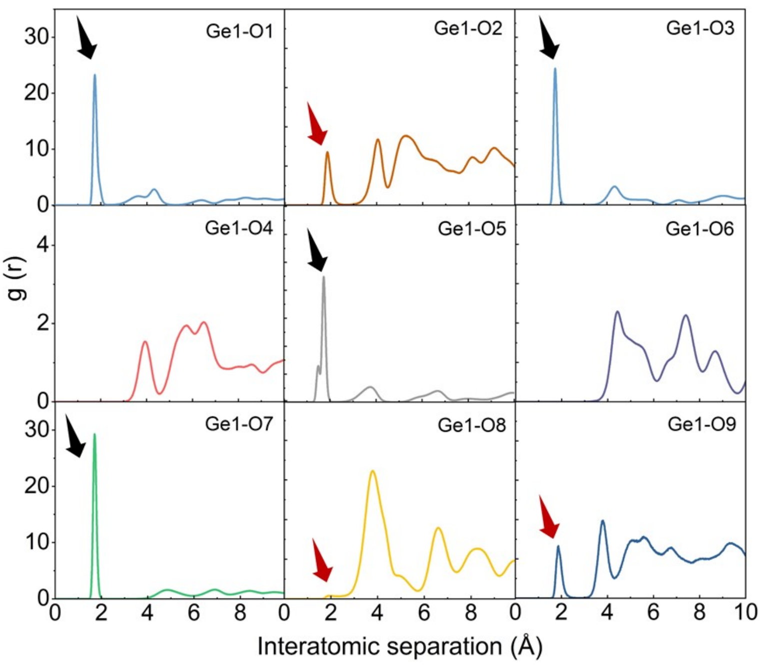

Figure S11. RDFs of Ge1-O interactions with the simulation time within the Ga1Ge2 layer, where the black and red arrows denote the interactions of Ge1 with the original site O (O1, O3, O5 and O7) and the derivative site O (O2 and O9), respectively.

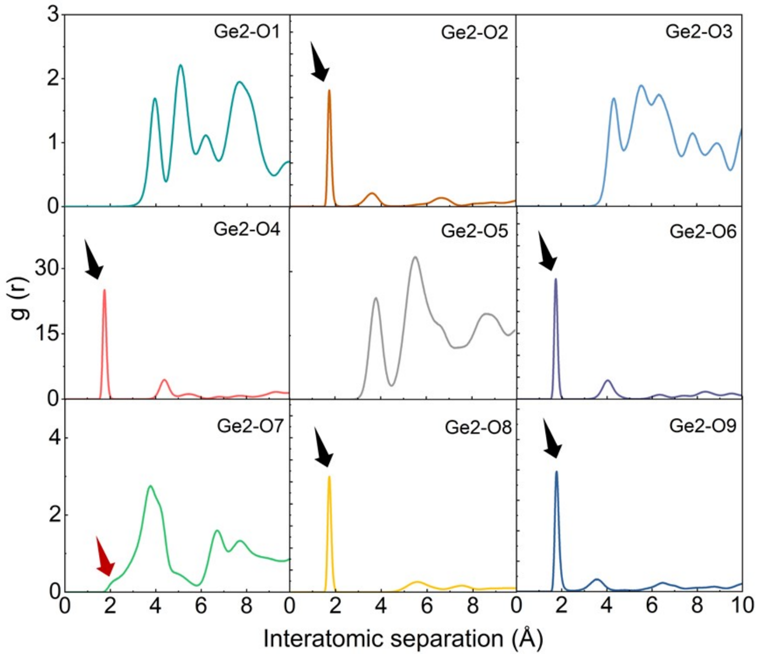

Figure S12. RDFs of Ge2-O interactions with the simulation time within the Ga1Ge2 layer, where the black arrows denote the interactions of Ge2 with the original site O (O2, O4, O6, O8 and O9).

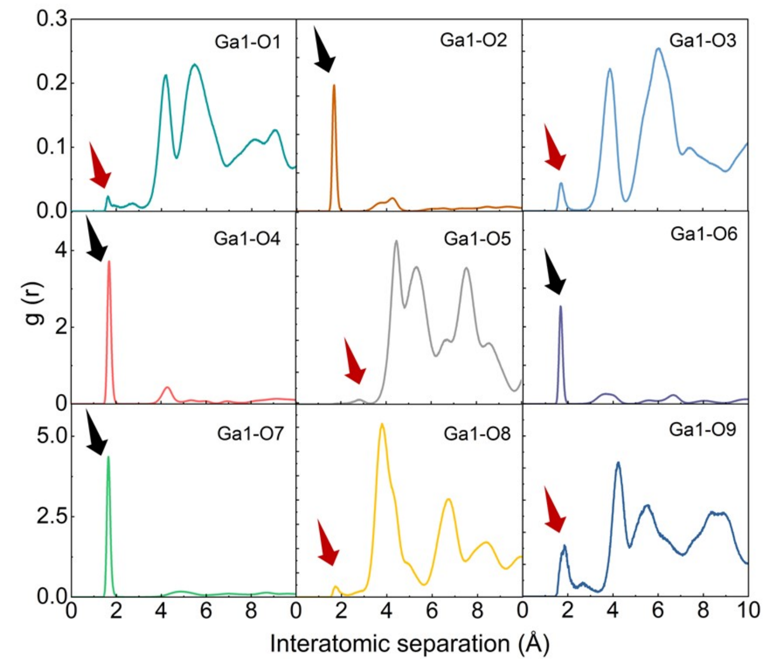

Figure S13. RDFs of Ga1-O interactions with the simulation time within the Ga2Ge1 layer, where the black and red arrows denote the interactions of Ga1 with the original site O (O2, O4, O6 and O7) and the derivative site O (O1, O3, O5, O8 and O9), respectively.

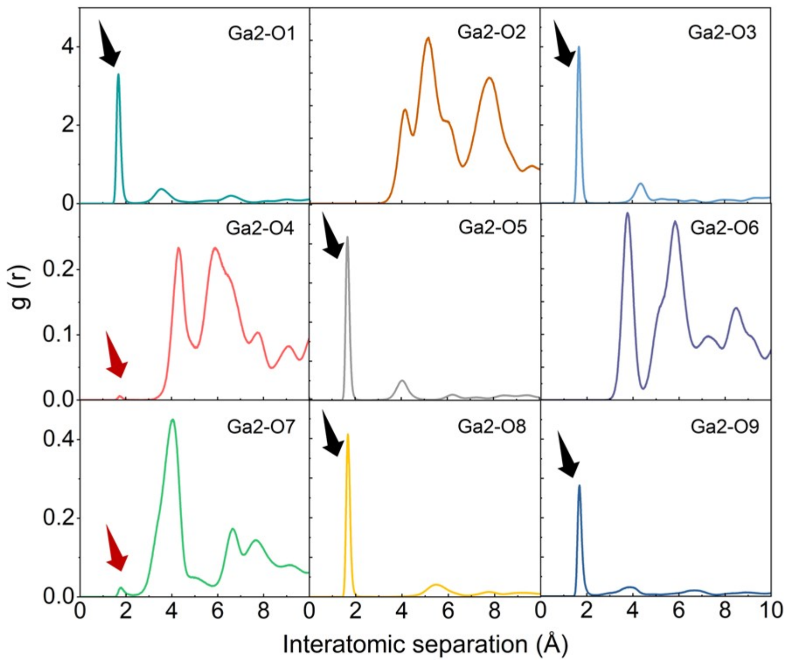

Figure S14. RDFs of Ga2-O interactions with the simulation time within the Ga2Ge1 layer, where the black and red arrows denote the interactions of Ga2 with the original site O (O1, O3, O5, O8 and O9) and the derivative site O (O2 and O7), respectively.

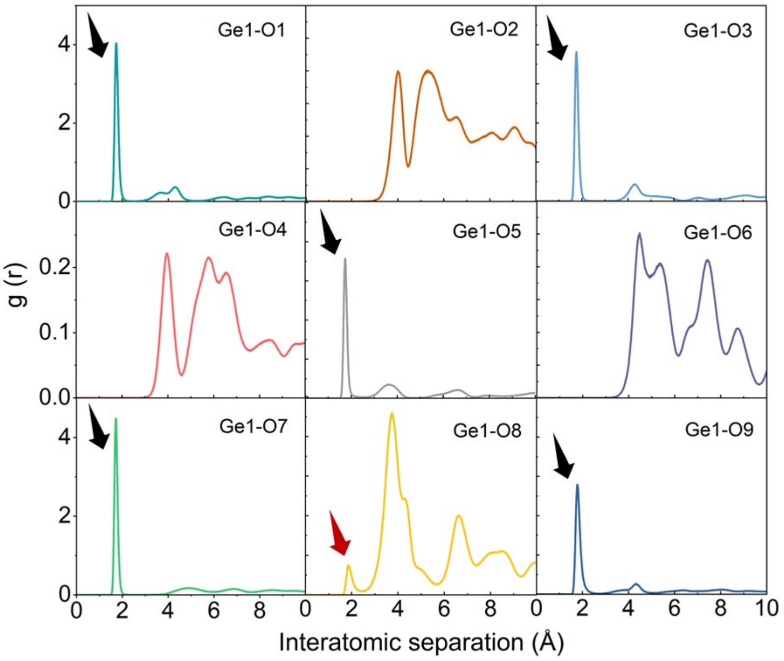

Figure S15. RDFs of Ge1-O interactions with the simulation time within the Ga2Ge1 layer, where the black and red arrows denote the interactions of Ge1 with the original site O (O1, O3, O5, O7 and O9) and the derivative site O (O8), respectively.

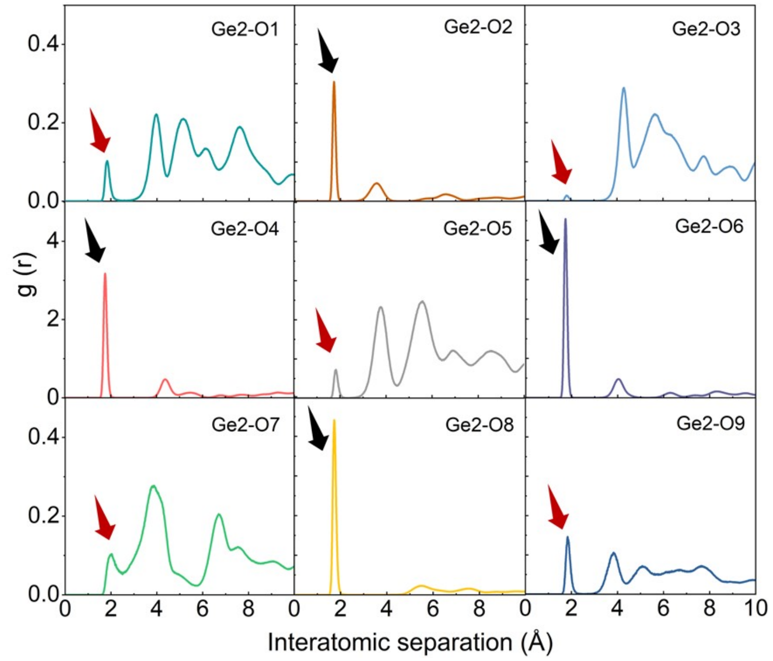

Figure S16. RDFs of Ge2-O interactions with the simulation time within the Ga2Ge1 layer, where the black and red arrows denote the interactions of Ge2 with the original site O (O2, O4, O6 and O8) and the derivative site O (O1, O3, O5, O7 and O9), respectively.
